# Protective effect of rosiglitazone on kidney function in high-fat challenged human-CRP transgenic mice: a possible role for adiponectin and miR-21?

**DOI:** 10.1038/s41598-017-02444-2

**Published:** 2017-06-06

**Authors:** Martine C. Morrison, Gopala K. Yakala, Wen Liang, Peter Y. Wielinga, Kanita Salic, Arianne van Koppen, Tushar Tomar, Robert Kleemann, Peter Heeringa, Teake Kooistra

**Affiliations:** 1 Department of Metabolic Health Research, Netherlands Organization for Applied Scientific Research (TNO), Zernikedreef 9, 2333 CK, Leiden The Netherlands; 2 Department of Pathology and Medical Biology, University of Groningen, University Medical Center Groningen, Hanzeplein 1 (EA11), 9713 GZ, Groningen The Netherlands

## Abstract

Obesity-related albuminuria is associated with decline of kidney function and is considered a first sign of diabetic nephropathy. Suggested factors linking obesity to kidney dysfunction include low-grade inflammation, insulin resistance and adipokine dysregulation. Here, we investigated the effects of two pharmacological compounds with established anti-inflammatory properties, rosiglitazone and rosuvastatin, on kidney dysfunction during high-fat diet (HFD)-induced obesity. For this, human CRP transgenic mice were fed standard chow, a lard-based HFD, HFD+rosuvastatin or HFD+rosiglitazone for 42 weeks to study effects on insulin resistance; plasma inflammatory markers and adipokines; and renal pathology. Rosiglitazone but not rosuvastatin prevented HFD-induced albuminuria and renal fibrosis and inflammation. Also, rosiglitazone prevented HFD-induced KIM-1 expression, while levels were doubled with rosuvastatin. This was mirrored by miR-21 expression, which plays a role in fibrosis and is associated with renal dysfunction. Plasma insulin did not correlate with albuminuria. Only rosiglitazone increased circulating adiponectin concentrations. In all, HFD-induced albuminuria, and renal inflammation, injury and fibrosis is prevented by rosiglitazone but not by rosuvastatin. These beneficial effects of rosiglitazone are linked to lowered miR-21 expression but not connected with the selectively enhanced plasma adiponectin levels observed in rosiglitazone-treated animals.

## Introduction

Obesity rates are rapidly rising worldwide in almost all populations and age groups, largely due to increased availability and consumption of calorie-dense foods with a high-fat, high-sugar content and lack of physical activity^1^. Obesity-related fat accumulation, especially in visceral depots, is associated with an increased risk of a number of pathologies, including insulin resistance (IR)^2^ and (micro) albuminuria^3^. Albuminuria has been associated with a decline of kidney function and is now being recognised not only as an important risk factor for future cardiovascular events^4, 5^, but is also considered a first sign of diabetic nephropathy^6^. With the recent rise in the prevalence of obesity, there is an urgent need for a better understanding of why a relationship exists between obesity and albuminuria and how obesity-related albuminuria develops. Suggested factors linking obesity to albuminuria include systemic chronic low-grade inflammation, IR, and specific adipocyte-derived adipokines.

Chronic low-grade inflammation, as evidenced by elevated plasma levels of acute-phase inflammatory markers, including C-reactive protein (CRP), a commonly used marker for systemic inflammation in humans^7^, is thought to play an important role in the development of both IR and nephropathy. To show a causative relationship between these risk markers and kidney disease, an intervention directed at reduction of systemic inflammation should in turn at least partly diminish IR and prevent albuminuria. If that were true, interventions that reduce systemic inflammation and insulin are attractive candidates for preventive treatment of patients at risk for developing (diabetic) nephropathy.

Another explanation for renal disease in obesity may be related to the notion that adipocytes are an active endocrine cell type^8, 9^. Adipocytes secrete several bioactive factors (adipokines) that reportedly play a role in maintaining metabolic health (reviewed in ref. 8). Obesity frequently leads to a dysregulation of adipokine secretion from fat depots^8^ and thus may be associated with metabolic diseases. Of the numerous factors that are regulated with increased visceral obesity, one of the best characterised is adiponectin. Recent clinical studies suggest that lowered plasma levels of adiponectin may play a key role in the development of obesity-related albuminuria^10^. Adiponectin is thought to regulate the function of podocytes, a renal cell-type that plays a significant role in the glomerular filtration barrier^11^. Indeed, studies in adiponectin knockout mice indicate that absence of adiponectin can contribute to the initial development of albuminuria^10^. Further evidence for beneficial effects of adiponectin on kidney functioning was sought by increasing plasma levels by administration of exogenous adiponectin, but these efforts were hampered by inherent difficulties in producing functional recombinant adiponectin, combined with the brief circulating half-life of adiponectin^12^. Therefore, efforts to increase adiponectin levels have also been focused on increasing the production of endogenous adiponectin by adipose tissue. Since the human and mouse adiponectin promoter contains binding sites for peroxisome proliferator-activated receptor gamma (PPAR-γ), pharmacological activation of PPAR-γ offers the opportunity to enhance endogenous plasma levels of adiponectin and thereby to further substantiate a protective role of adiponectin in the development of kidney disease.

To gain more insight into the role of inflammation and adiponectin in metabolic-stress-induced albuminuria, renal inflammation and fibrosis in the context of IR, we used a human CRP transgenic (huCRPtg) mouse model. The huCRPtg mouse carries a transgene containing the human CRP gene, the 5′ flanking promoter region and all known human CRP gene regulatory elements^13^. These mice have been successfully employed to monitor systemic inflammation and to determine the effects and mechanisms of drugs like statins and fibrates in reducing inflammatory process^14^. In a recent study^15^, we demonstrated that by feeding a high-fat diet (HFD), huCRPtg mice showed metabolic-stress-induced systemic inflammation and developed osteoarthritis. Interventions with a statin (rosuvastatin) and a PPAR-γ activator (rosiglitazone) reduced systemic inflammation as indicated by decreased human CRP levels and concomitantly inhibited the development of osteoarthritis. Here we have used this mouse model to evaluate whether suppression of HFD-induced systemic inflammation by rosuvastatin and rosiglitazone also improves albuminuria, renal inflammation and fibrosis under conditions of obesity and IR. An integral part of the study was to assess a putative role of adiponectin, which is induced by rosiglitazone.

## Results

### Body weight and fat distribution

HuCRPtg mice were fed a standard chow diet, a lard-based HFD, HFD+0.005% rosuvastatin or HFD+0.018% rosiglitazone for 42 weeks to study effects on insulin resistance, plasma inflammatory markers and adipokines, and renal pathology (albuminuria, inflammation and fibrosis). Body weight at baseline (t = 0) was 28.5 ± 2.1 g. The three experimental groups that received HFD all showed a gradual increase in body weight over time and all had significantly higher average body weights at the end of the experimental period (t = 42 weeks) compared with the group that remained on chow (35.1 ± 1.9 g). Mice treated with rosiglitazone had the highest body weight (57.7 ± 11.0 g), which was significantly higher than that of the HFD group (41.1 ± 4.7 g) and the rosuvastatin-treated group (46.1 ± 6.2 g)^15^.

**Figure 1:**
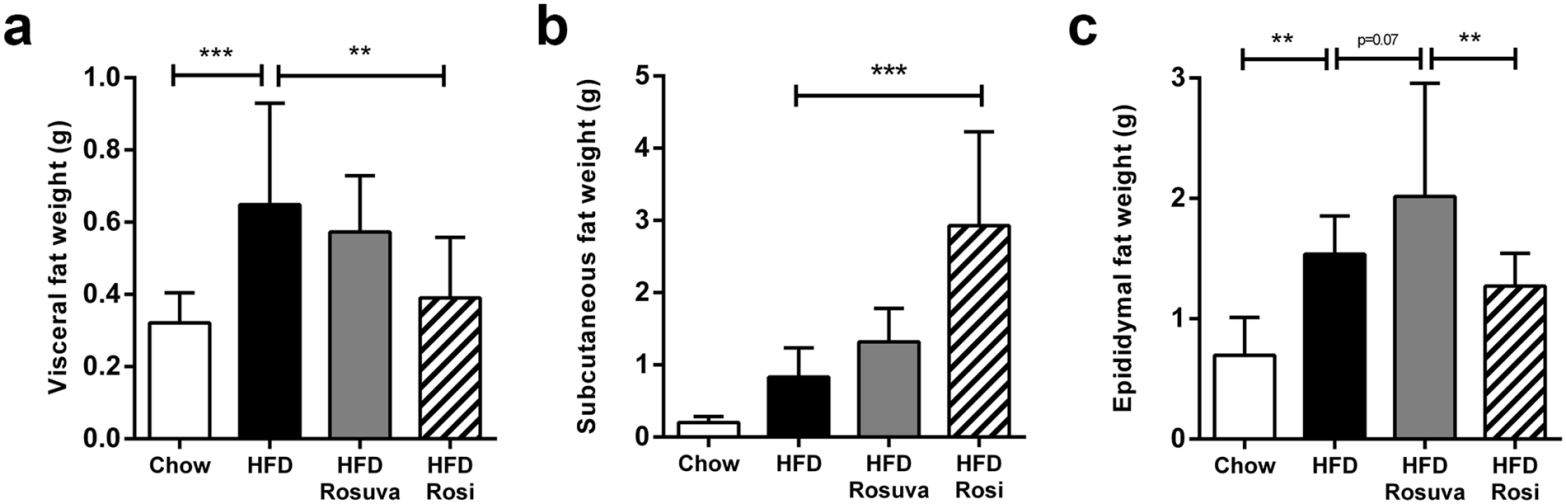
Effects of rosuvastatin and rosiglitazone on fat distribution. HuCRPtg mice were fed chow, high-fat diet (HFD), HFD+0.005% (w/w) rosuvastatin (HFD Rosuva) or HFD+0.018% (w/w) rosiglitazone (HFD Rosi) for 42 weeks. (**a**) Visceral fat mass, (**b**) subcutaneous fat mass, (**c**) epididymal fat mass. Data are mean ± SD. **p < 0.01, ***p < 0.001.

All mice fed HFD showed a clear increase in fat mass compared with chow-fed mice, but what was most striking were the observed differences in fat mass distribution over the various fat depots in the different groups (Fig. 1a–c). Rosiglitazone treatment resulted in a reduction in visceral fat mass and an increase in subcutaneous fat mass in comparison with the HFD group (visceral fat: 0.39 ± 0.17 g vs. 0.65 ± 0.29 g, p < 0.01; subcutaneous fat: 2.93 ± 1.30 g vs. 0.83 ± 0.41 g, p < 0.001). In the rosuvastatin group, an increase in epididymal fat mass was observed in comparison with both the HFD group (HFD+Rosuva vs. HFD, 2.02 ± 0.95 g vs. 1.54 ± 0.32 g, p = 0.07) and the rosiglitazone group (HFD+Rosuva vs. HFD+Rosi, 2.02 ± 0.95 g vs. 1.27 ± 0.28 g, p < 0.01).

### Glucose and insulin levels

**Figure 2:**
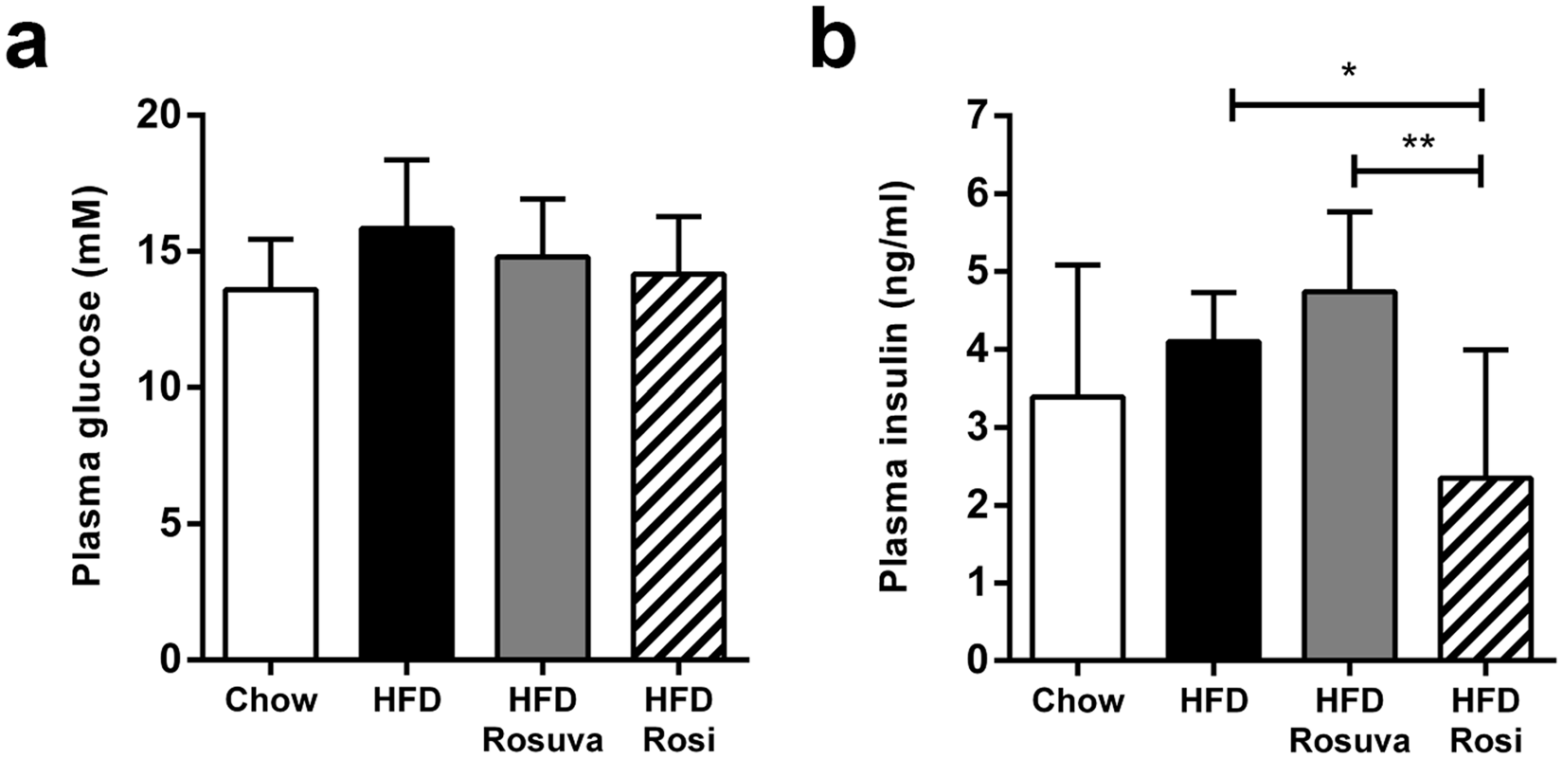
Effects of rosuvastatin and rosiglitazone on plasma glucose and insulin. HuCRPtg mice were fed chow, high-fat diet (HFD), HFD+0.005% (w/w) rosuvastatin (HFD Rosuva) or HFD+0.018% (w/w) rosiglitazone (HFD Rosi) for 42 weeks. (**a**) Plasma glucose levels, (**b**) plasma insulin levels. Data are mean ± SD. *p < 0.05, **p < 0.01.

Plasma glucose levels were 9.8 ± 1.21 mM at the start of the experiment and increased steadily and significantly during the rest of the investigational period for all experimental groups: 13.6 ± 1.8 mM (chow), 15.9 ± 2.5 mM (HFD), 14.8 ± 2.1 mM (HFD+Rosuva), and 14.2 ± 2.1 mM (HFD+Rosi) at t = 42 (Fig. 2a). Compared with glucose levels, changes in insulin levels over time were more pronounced and differed between groups. Average insulin levels were 0.6 ± 0.3 ng/ml at t = 0 and rose over time to 3.4 ± 1.7 ng/ml at t = 42 weeks for the chow group and to 4.1 ± 0.6 ng/ml in HFD. Notably, the highest insulin levels were observed with rosuvastatin treatment (4.7 ± 1.0 ng/ml), whereas rosiglitazone treatment markedly and significantly suppressed insulin levels to 2.3 ± 1.6 ng/ml (p < 0.05 compared with HFD) (Fig. 2b).

### Plasma adipokines

**Figure 3:**
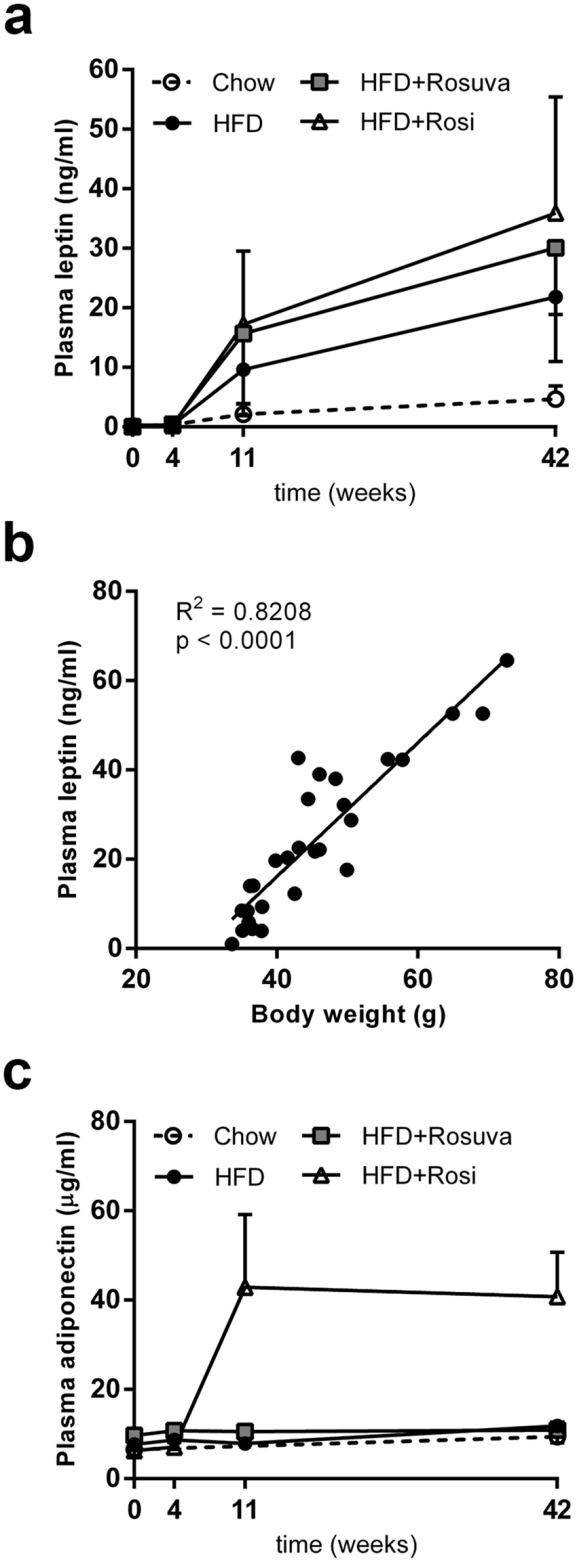
Effects of rosuvastatin and rosiglitazone on plasma leptin and adiponectin levels. HuCRPtg mice were fed chow, high-fat diet (HFD), HFD+0.005% (w/w) rosuvastatin (HFD Rosuva) or HFD+0.018% (w/w) rosiglitazone (HFD Rosi) for 42 weeks. (**a**) Plasma leptin levels over time. (**b**) Correlation between plasma leptin and body weight. (**c**) Plasma adiponectin levels over time. Data are mean ± SD.

Leptin is an adipokine that is highly specific for adipose tissue. Several studies of obese humans have shown a strong and consistent positive relation between plasma leptin concentrations and adipose tissue mass (see ref. 16 and references therein). At the start of the experiment, plasma leptin levels were low (0.15 ± 0.3 ng/ml) and they increased slightly over time in the chow group (4.7 ± 2.2 ng/ml at t = 42 weeks) (Fig. 3a). After starting the HFD, leptin levels gradually and strongly rose in all three HFD groups (Fig. 3a). At t = 42 weeks, leptin levels in the rosiglitazone group had reached values of 35.9 ± 19.5 ng/ml, higher than those of the rosuvastatin group (30.1 ± 11.2 ng/ml) and the HFD group (21.8 ± 10.9 ng/ml, p < 0.05). Correlation analysis revealed a strong positive relationship between body mass and leptin levels (R^2^ = 0.8208, p < 0.0001, Fig. 3b), in line with the reported correlation between plasma leptin levels and adipose mass in humans^16^. In contrast to leptin, adiponectin levels are usually reduced with increasing obesity and associated comorbidities, such as type-2 diabetes (T2D)^17^. Grosso modo, adiponectin levels remained relatively constant in all treatment groups during the entire experimental period (t = 0: 7.4 ± 2.5 µg/ml; and t = 42: 9.4 ± 3.3 µg/ml in chow, 11.9 ± 1.2 µg/ml in HFD, and 10.9 ± 2.8 µg/ml in HFD+Rosuva), except for the rosiglitazone group, which showed strongly increased plasma concentrations of adiponectin from t = 4 weeks onward, reaching average levels of 40.8 ± 9.9 µg/ml (p < 0.001 compared with all other groups at t = 42, Fig. 3c).

### Renal inflammation and renal function

**Figure 4:**
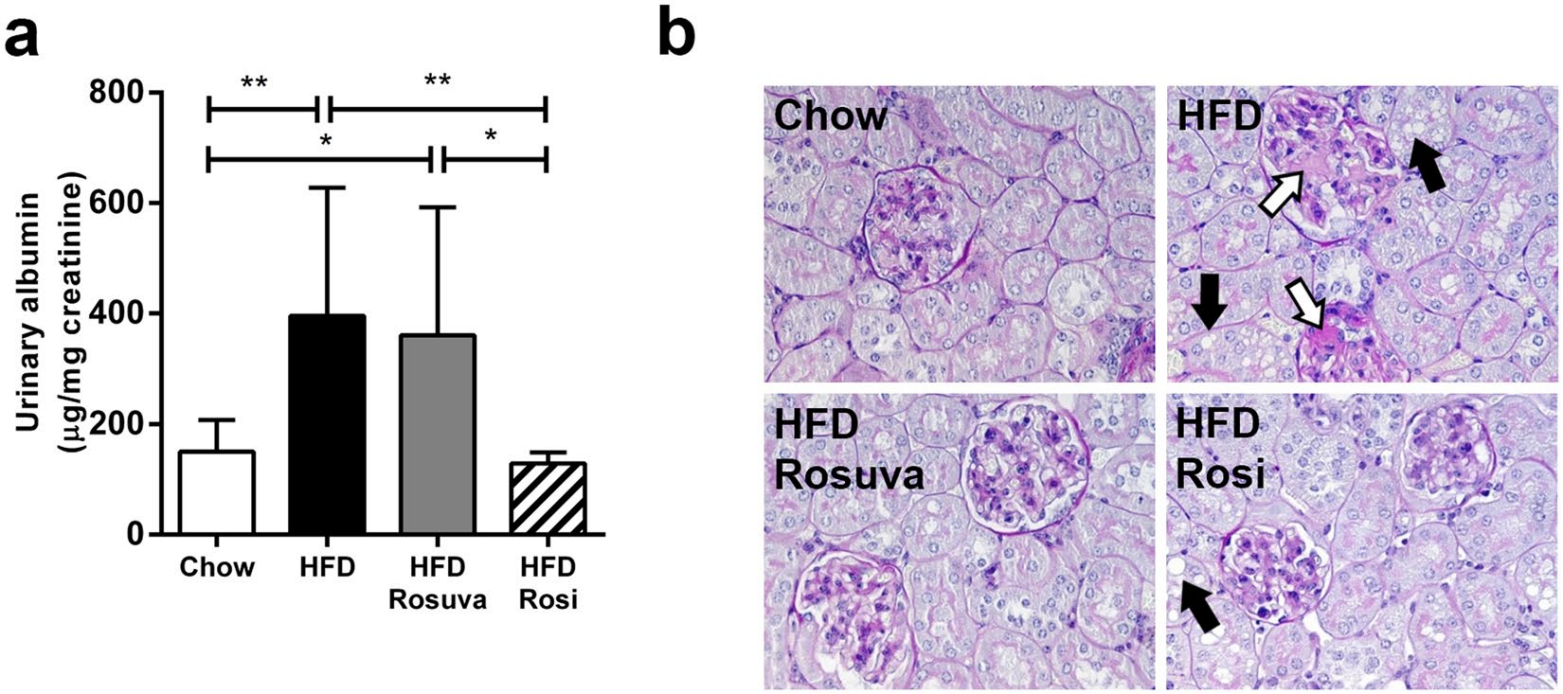
Effects of rosuvastatin and rosiglitazone on urinary albumin and kidney histology. HuCRPtg mice were fed chow, high-fat diet (HFD), HFD+0.005% (w/w) rosuvastatin (HFD Rosuva) or HFD+0.018% (w/w) rosiglitazone (HFD Rosi) for 42 weeks. (**a**) Urinary albumin levels. (**b**) Representative photomicrographs of PAS-stained kidney sections showing mesangial expansion (open arrows) and lipid droplets in tubuli (filled arrows). Data are mean ± SD., *p < 0.05, **p < 0.01.

At the end of the experimental period, the chow group had a urinary albumin/creatinine ratio of 151 ± 57 µg/mg. Both the HFD group (396 ± 232 µg/mg, p < 0.01) and the Rosuva group (361 ± 232 µg/mg, p < 0.05) showed a comparable and significant increase in the albumin/creatinine ratio compared with the chow group (Fig. 4a). In contrast, HFD-fed mice treated with rosiglitazone exhibited urinary albumin/creatinine ratios of 129 ± 20 µg/mg, i.e. similar to those of chow-fed mice, and well below the values seen for the HFD and Rosuva groups (p < 0.01 compared with HFD, p < 0.05 compared with HFD+Rosuva; Fig. 4a). Notably, urinary albumin levels were not significantly correlated with plasma insulin levels (not shown).

Bright-field microscopy analysis revealed that HFD feeding induced development of mild mesangial area expansion and accumulation of lipid droplets in tubuli (Fig. 4b). Both rosuvastatin and rosiglitazone prevented mesangial expansion. Tubular lipid accumulation was also observed in the rosiglitazone-treated group, but was absent in the rosuvastatin-treated mice.

Gene expression analysis revealed that relative mRNA expression levels (expressed as fold-change relative to chow) of kidney injury molecule 1 (KIM-1) were markedly upregulated in HFD (2.15 ± 0.84, p = 0.08 vs. chow, Table 1). Notably, rosuvastatin markedly and significantly further increased KIM-1 expression levels (3.56 ± 2.18, p < 0.05 vs. HFD), whereas rosiglitazone kept KIM-1 mRNA levels as low as those found for the chow group, i.e. strikingly below values seen for the HFD and HFD+Rosuva groups. Immunofluorescence staining of KIM-1 confirmed mild HFD-induced damage around tubular cells, showing a limited number of positively stained tubuli, which was absent in chow controls (not shown).

**Table 1:** Effects of rosiglitazone and rosuvastatin on renal mRNA expression.

	Chow	HFD	HFD+Rosuva	HFD+Rosi
KIM-1	1.00 ± 0.25^a^	2.15 ± 0.84^ac^	3.56 ± 2.18^b^	1.22 ± 0.55^ac^
E-selectin	1.00 ± 0.33^a^	2.41 ± 0.73^b^	2.23 ± 0.85^b^	1.38 ± 0.54^a^
CD68	1.00 ± 0.13^a^	1.20 ± 0.33^ab^	1.31 ± 0.23^b^	1.41 ± 0.34^b^
VCAM-1	1.00 ± 0.22^a^	0.83 ± 0.31^a^	1.11 ± 0.38^a^	0.94 ± 0.27^a^

Fold-change relative to chow. Data are mean ± SD. Means in a row with superscripts that do not share a common letter differ significantly (p < 0.05).

A similar pattern emerged with respect to the renal mRNA expression levels of the endothelial activation marker E-selectin (Table 1). HFD feeding significantly upregulated E-selectin mRNA expression (2.41 ± 0.73, p < 0.001 vs. chow), and rosuvastatin was unable to prevent this increase (2.23 ± 0.85, n.s. vs. HFD). In contrast, rosiglitazone treatment considerably downregulated E-selectin mRNA expression (1.38 ± 0.54, p < 0.01 vs. HFD), with expression levels similar to those observed in chow-fed mice.

The expression of CD68 mRNA, a marker for macrophage infiltration, and VCAM-1 mRNA, a vascular endothelial activation marker, did not differ significantly between the experimental groups (Table 1).

### Plasma markers of systemic inflammation

**Figure 5:**
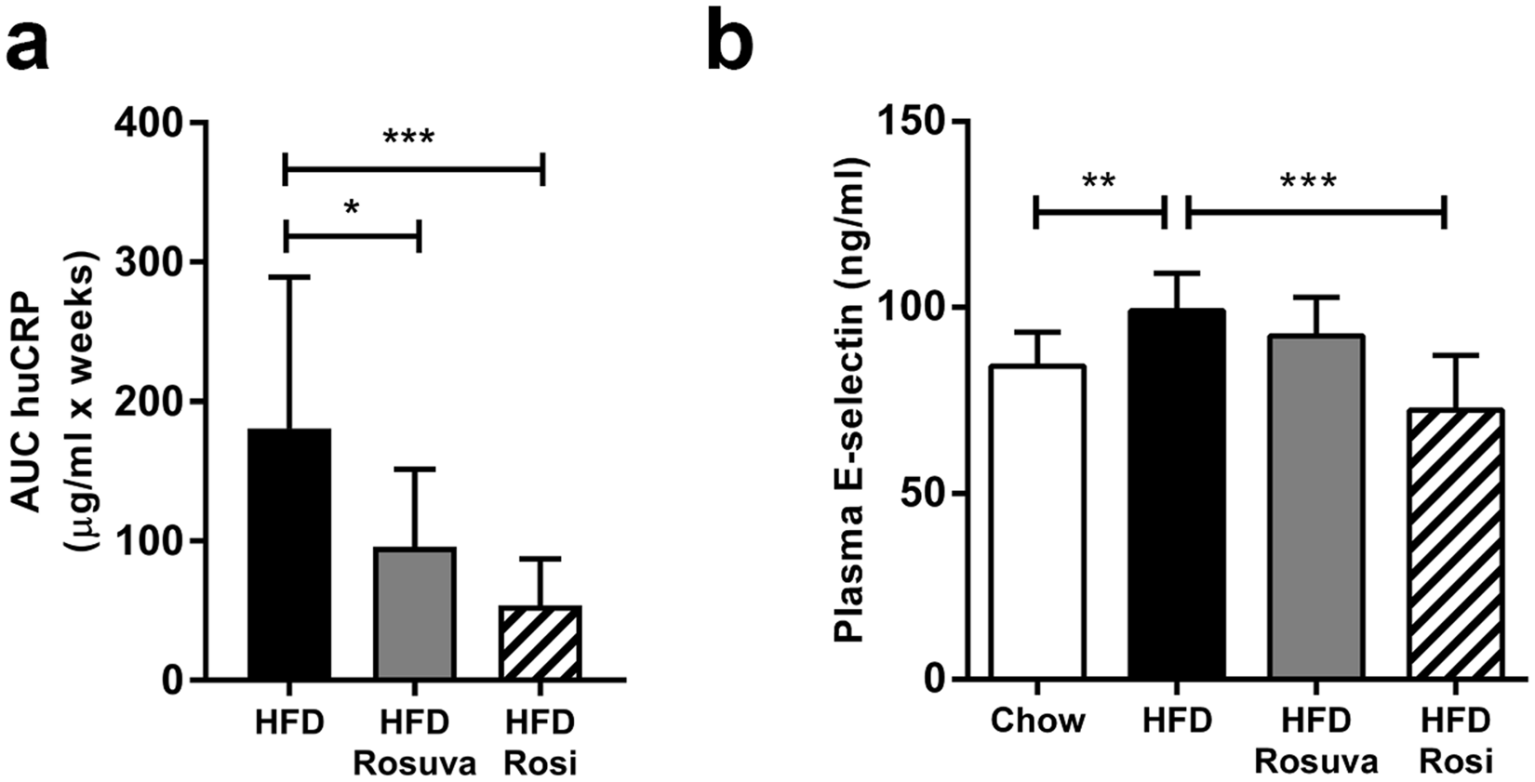
Effects of rosuvastatin and rosiglitazone on plasma huCRP and E-selectin. HuCRPtg mice were fed chow, high-fat diet (HFD), HFD+0.005% (w/w) rosuvastatin (HFD Rosuva) or HFD+0.018% (w/w) rosiglitazone (HFD Rosi) for 42 weeks. (**a**) huCRP exposure during HFD-feeding (**b**) plasma E-selectin at t = 42 weeks. Data are mean ± SD. **p < 0.01, ***p < 0.001.

HuCRP levels were measured to monitor the overall systemic inflammatory state induced by HFD, and the effect of interventions with rosuvastatin and rosiglitazone thereupon. As reported previously, plasma huCRP levels were increased by HFD feeding and this induction was significantly quenched in animals treated with rosiglitazone or rosuvastatin^15^, as is also reflected by the significant reduction in huCRP exposure during HFD-feeding (Fig. 5a). In contrast, only rosiglitazone markedly and significantly reduced the HFD-induced increase in plasma E-selectin levels at 42 weeks thus reflecting the renal mRNA data for E-selectin (Fig. 5b).

### Renal fibrosis and miR-21

**Figure 6:**
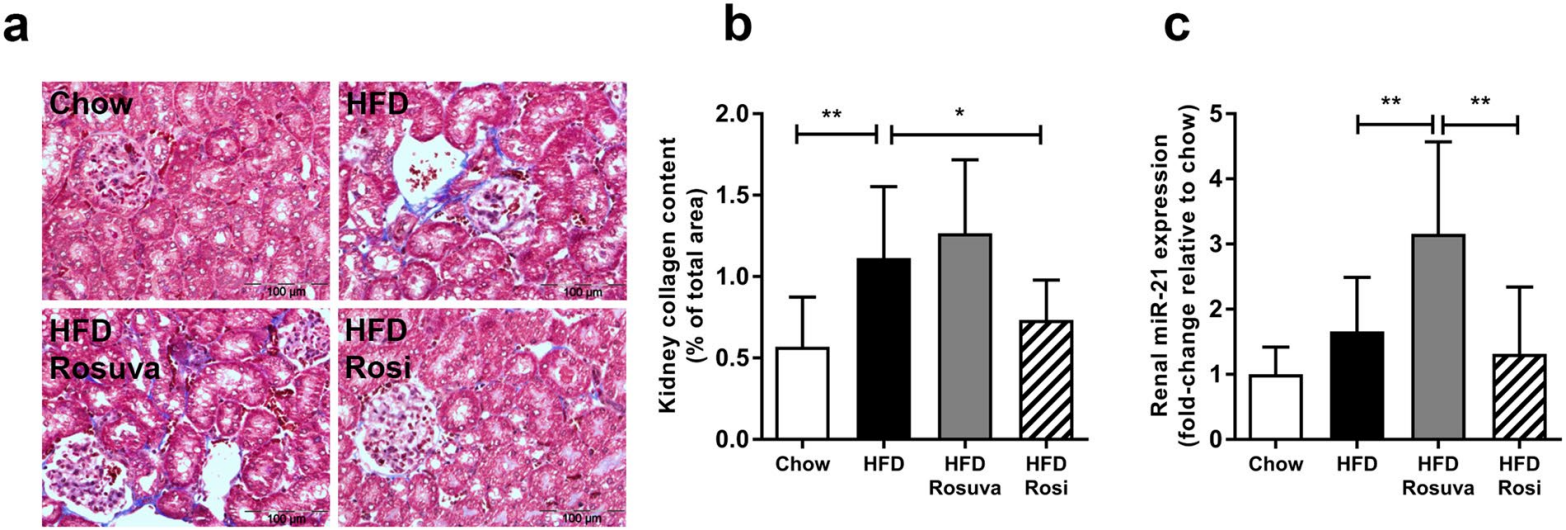
Effects of rosuvastatin and rosiglitazone on kidney fibrosis. HuCRPtg mice were fed chow, high-fat diet (HFD), HFD+0.005% (w/w) rosuvastatin (HFD Rosuva) or HFD+0.018% (w/w) rosiglitazone for 42 weeks. (**a**) Representative photomicrographs of kidney sections stained with Masson’s Trichrome showing renal collagen deposition. (**b**) Quantification of tubular interstitial collagen (Sirius Red staining quantified by ImageJ analysis). (**c**) Renal miR-21 expression. Data are mean ± SD. *p < 0.05, **p < 0.01.

Renal fibrosis is a frequent underlying cause of decreased renal function. To gain insight into fibrosis development, kidneys were stained with Masson Trichrome and Picro-Sirius Red, and analyzed for collagen deposition. Figure 6a shows Masson’s Trichrome staining with the corresponding collagen quantification (as quantified in Picro-Sirius Red-stained sections) in Fig. 6b. HFD induced the tubular interstitial collagen content (1.11 ± 0.44%) compared with the chow group (0.57 ± 0.31%; p < 0.01, Fig. 6b). Notably, while collagen content after rosuvastatin treatment was comparable to that in the HFD group (1.27 ± 0.45%), rosiglitazone significantly prevented collagen deposition (0.74 ± 0.24%; p < 0.05 compared with HFD) with levels comparable to those in the chow group.

Since miR-21 has been shown to play a pathological role in many forms of fibrosis and since its increase is associated with microalbuminuria, inflammation and renal fibrosis^18, 19^, we examined renal miR-21 expression (Fig. 6c). Renal miR-21 expression was enhanced in HFD compared with chow group (fold-change 1.66 ± 0.83 in HFD vs., 1.00 ± 0.42 in chow). Rosuvastatin treatment nearly doubled miR-21 expression (fold-change 3.16 ± 1.41 relative to chow; p < 0.01 compared with HFD) on top of HFD treatment. In contrast, rosiglitazone kept miR-21 levels low (fold-change 1.32 ± 1.02 relative to chow). To test whether miR-21 expression is connected to kidney pathology, we performed correlation analyses. Renal miR-21 expression correlated significantly with renal KIM-1 expression (Spearman r = 0.46, p = 0.02) and renal fibrosis (Spearman r = 0.52, p = 0.003) while no such correlations were found for plasma adiponectin levels or adiponectin exposure.

## Discussion

Obesity-related albuminuria is recognised as a first sign of declined kidney function. Among the factors suggested to connect obesity to kidney dysfunction are low-grade systemic inflammation, IR/T2D and adipokine dysregulation. In the current study we sought evidence for the role of these obesity-linked factors in the development of aspects of renal pathology, viz. albuminuria, inflammation and fibrosis. For this, we employed huCRPtg mice under conditions of HFD-induced obesity in conjunction with two pharmacological interventions, rosiglitazone and rosuvastatin, with established anti-inflammatory properties^15^, as exemplified by decreased huCRP levels. Our results demonstrate that anti-inflammatory rosiglitazone but not anti-inflammatory rosuvastatin prevented HFD-induced albuminuria and renal fibrosis, and inhibited expression of the renal inflammation marker, E-selectin. Rosiglitazone also prevented the HFD-enhanced mRNA expression of KIM-1, while rosuvastatin almost doubled KIM-1 mRNA levels. Notably, these beneficial effects of rosiglitazone were paralleled by absence of miR-21 induction, the expression of which was found to be correlated with kidney pathology (i.e. KIM-1 expression and kidney fibrosis).

In our study, we focused on human CRP levels as a marker for systemic inflammation. Recently, it has been reported that rosiglitazone treatment reduces plasma CRP levels in rats with streptozotocin-induced T2D^20^. Consistent with these findings, we observed that rosiglitazone treatment reduced human CRP levels in HFD-challenged huCRPtg mice indicating that rosiglitazone suppresses systemic inflammatory responses^15^. Rosiglitazone is a PPAR-γ agonist that belongs to the thiazolidinedione class of drugs. It has been reported that in patients with T2D, treatment with another thiazolidinedione (pioglitazone) along with insulin therapy decreased human CRP levels when compared to insulin treatment alone^21, 22^. In the current study, rosiglitazone reduced plasma insulin levels and hence reduced IR. In contrast, anti-inflammatory rosuvastatin treatment^15^ failed to reduce circulating insulin levels, which is in line with a recent study showing that rosuvastatin failed to improve IR in patients on peritoneal dialysis^23^.

Rosiglitazone treatment caused a significant increase in body weight in huCRPtg mice compared with HFD mice. More specifically, rosiglitazone-treated mice especially had increased subcutaneous fat mass compared with HFD mice. Consistent with these observations, treatment with PPAR-γ agonists has been shown to improve insulin sensitivity despite increasing body fat mass, in particular subcutaneously^21^. In contrast, an increase in visceral fat has been reported to be highly associated with IR and T2D^22, 23^. Here, we observed that rosiglitazone treatment decreased visceral fat mass. In contrast, in rosuvastatin-treated mice the mass and distribution of the fat depots were similar to those observed in untreated HFD-challenged mice.

We also observed that mice treated with rosiglitazone displayed markedly increased plasma levels of adiponectin, a protein predominantly secreted by adipocytes. It is well documented that adiponectin is a cardioprotective adipokine, due to its anti-inflammatory and insulin-sensitizing properties^24, 25^. An inverse relationship between adiponectin levels and CVD has been reported^15, 26^ in patients with end-stage renal disease, and there is increasing evidence that adiponectin plays a protective role in T2D and IR. For example, adiponectin-deficient mice are prone to develop IR and vascular damage after HFD challenge^27^ whereas treatment with adiponectin inhibits renal fibrosis and albuminuria in adiponectin knock-out mice^24^. Moreover, enhanced expression of adiponectin attenuates inflammation and diabetes development in db/db mice^28^. Since plasma levels of adiponectin and adiponectin exposure over time did not correlate with kidney disease severity (percentage of fibrosis and KIM-1 expression) in the present study it is unlikely that adiponectin plays a causal role under the experimental conditions applied herein.

Obesity-related albuminuria is now being recognised not only as an indication of declined kidney function and a first sign of diabetic nephropathy^10^, but also increases the risk of CVD^29^. If left untreated, patients with albuminuria are more prone to CVD. Treatments that reduce albuminuria are therefore inherently renoprotective and would improve CVD outcomes^29^. We observed that mice on HFD developed mesangial expansion, lipid accumulation and albuminuria. Furthermore, KIM-1, a marker of kidney injury^30^, was upregulated in mice on HFD. Rosiglitazone treatment markedly diminished these HFD-induced renal effects and improved kidney function as evidenced by reduced urinary albumin/creatinine ratios and lowered KIM-1 expression. Moreover, rosiglitazone treatment strikingly reduced plasma E-selectin levels and, similarly, reduced renal E-selectin mRNA expression levels. Conversely, rosuvastatin treatment failed to exert any beneficial effects on markers of endothelial activation and inflammation in the kidney and did not improve albumin/creatinine ratios. In fact, the expression levels of some markers, including E-selectin and KIM-1 were increased in the kidneys of rosuvastatin-treated mice when compared with those of chow and HFD-challenged mice.

In a healthy individual, the kidney is able to reabsorb the majority of protein that enters the renal filtrate, with only traces being excreted in the urine. In the obese state there are two main sites involving two different processes that result in a loss of protein (mainly albumin) in the urine, viz. (i) structural changes to the glomerulus allowing more albumin to enter the filtrate and (ii) inability of the proximal tubules to endocytose the increased protein load (see ref. 9 and references therein). The development of albuminuria is a typical characteristic of renal damage (nephropathy ref. 31). Previous studies have demonstrated that statins can inhibit tubular reabsorption of filtered albumin^32, 33^. Furthermore, this notion is supported by a recent study in hypertensive patients demonstrating that statin treatment was independently associated with the occurrence of microalbuminuria^34^. Exposure to raised levels of albumin in the renal tubule has been linked to increased proinflammatory and profibrotic changes in the tubulointerstitium^35^.

Diabetic patients with end-stage kidney disease have a 5-year survival rate of merely 20%^36^. One of the major features of diabetic nephropathy is the presence of fibrosis. We observed increased collagen content in the HFD group. Notably, rosiglitazone, but not rosuvastatin treatment, prevented the increase in renal collagen deposition, suggesting a possible link between renal fibrosis and renal function. MiR-21 is the most substantial miRNA involved in many fibrotic diseases and its expression is enhanced after initiation of myocardial and pulmonary fibrosis^37, 38^. MiR-21 levels are also enhanced in human kidney fibrosis^18, 19^. Experimental support for a causative role of miR-21 is provided by miR21-silencing experiments. Silencing of miR-21 by anti-miR-21 oligonucleotide treatment in a murine model of Alport nephropathy reduced glomerulosclerosis, interstitial fibrosis, tubular injury, and inflammation^39^. Similarly, silencing miR-21 in diabetic kidneys of db/db mice ameliorated albuminuria, inflammation and renal fibrosis^40^. We observed highly enhanced miR-21 levels only after rosuvastatin treatment, whereas rosiglitazone prevented its expression and was comparable to the chow group. Notably, gene expression of pro-fibrotic genes like α-Sma and Col1α1 (results not shown) was unaffected suggesting that miR-21 could be an early marker of the initiating fibrosis in the kidneys.

In conclusion, our results demonstrate that rosiglitazone reduced HFD-induced insulin levels, suppressed the systemic inflammatory response and protected mice from the development of albuminuria. Despite its anti-inflammatory properties, rosuvastatin failed to improve IR and renal function. Strikingly, the beneficial effects of rosiglitazone were paralleled by lowered renal expression levels of miR-21; an increase of miR-21 is associated with microalbuminuria development, inflammation and renal fibrosis. In all, our findings suggest that adiponectin does not play a major role in the disease-attenuating effect of rosiglitazone and indicate that the option of quenching miR-21 activity merits follow-up.

## Materials and Methods

### Animal experiments

Animal experiments were approved by an independent Committee on the Ethics of Animal Experiments (DEC-Zeist, The Netherlands) and were in compliance with European Community specifications regarding the use of laboratory animals. All sample materials and organs required for this study were obtained from TNO-Biosciences, Leiden. Minor parts of the results (in particular body weight development over time) were published previously, where indicated, but are presented here as well for clarity^15^.

HuCRPtg mice^13, 14^ on a C57BL/6 background were characterised by PCR and ELISA for huCRP expression. Mice were housed in groups under standard conditions with a 12-h light-dark cycle and had free access to water and food. Mice of 12 weeks of age were fed either standard lab chow (ssniff® R/M-H, ssniff Spezialdiäten, Soest, Germany) or chow supplemented with 0.01% (w/w) rosuvastatin (“Crestor®”, AstraZeneca, Zoetermeer, the Netherlands) (t = 0). After 4 weeks (t = 4) the diets were changed and mice were divided into 4 groups consisting of 9 mice per group. Mice that had been fed chow were randomly distributed into 3 groups. Group 1 consisted of control mice that remained on the standard chow diet. Group 2 was switched to a high-fat (lard) diet (HFD) (23.6% fat/45% kcal% fat D12451, Research Diets, New Brunswick, New Jersey) and mice in group 3 were fed HFD containing 0.018% (w/w) rosiglitazone (HFD+Rosi) (Avandia, GSK, London, United Kingdom). Group 4 consisted of mice that had been fed standard chow supplemented with 0.01% (w/w) rosuvastatin (AstraZeneca), and were switched after 4 weeks to HFD containing 0.005% (w/w) rosuvastatin (HFD+Rosuva). The reason to lower the dose of rosuvastatin is the increased absorption of rosuvastatin in the context of HFD-feeding. The rosuvastatin and rosiglitazone doses were based on their anti-inflammatory effects observed in previous studies^41, 42^. Mice on chow diet (group 1), mice on HFD without drugs (group 2) and drug-treated mice on HFD (groups 3 and 4) remained on their respective diets until the completion of the study at 54 weeks of age (t = 42).

Body weight monitoring and blood sampling by tail incision after 4 hours of fasting was done at t = 0 and weeks 2, 4, 5, 11, 34, and 42. Blood samples were collected in EDTA tubes (Sarstedt AG & Co, Nümbrecht, Germany) and centrifuged for 10 minutes at 6000 rpm, after which plasma was collected and immediately stored at −80 °C until use. Spot urine and serum (after heart puncture) was collected from all animals at the time of sacrifice (t = 42). Mice were sacrificed by CO_2_ asphyxiation after a 4-hour fast, and tissues were isolated and weighed. Left kidneys were fixed in formalin and embedded in paraffin, and right kidneys were snap frozen in liquid N_2_ and stored at −80 °C until further use.

### Plasma analyses

Human CRP in plasma was quantified by established ELISA (R&D Systems, Abingdon, United Kingdom) at t = 0 and weeks 2, 4, 5, 11, 34, and 42. Plasma levels of E-selectin (R&D Systems), leptin (R&D Systems), and adiponectin (R&D systems) were quantified up to t = 42 weeks. Plasma insulin levels were determined by ELISA (Mercodia, Uppsala, Sweden) and plasma glucose levels were determined by enzymatic assay (glucose hexokinase method, Instruchemie, the Netherlands).

### Renal RNA and miRNA extraction and gene expression analysis

Total RNA was extracted from 35 µm thin cryo-sections from kidney using RNeasy Plus Mini Kit (Qiagen N.V., Venlo, the Netherlands) according to the manufacturer’s instructions. Integrity of RNA was determined by agarose gel electrophoresis. RNA quantity (OD-260) and quality (OD-260/OD-280) were determined using a NanoDrop1000 UV-Vis spectrophotometer (NanoDrop Technologies, Rockland, DE, USA). Total RNA was reverse-transcribed using SuperScript® III Reverse Transcriptase (Invitrogen, Breda,the Netherlands) and random hexamer primers (Promega, Leiden, the Netherlands). TaqMan® Gene Expression Assays (Life Technologies, Bleiswijk, the Netherlands) were used to detect the expression of selected target genes. PPIA (Mm02342430_g1) was used as an endogenous control along with the following probes for CD68 (Mm00839636_g1), VCAM-1 (Mm00449197_m1), E-selectin (Mm00441278_m1), and Kidney Injury Molecule-1 (KIM-1, Mm00506686_m1). Real-time PCR was performed in duplicate and the obtained threshold cycle (Ct) values were averaged. Relative mRNA levels were calculated using the comparative Ct (ΔΔCt) method, expressed as fold-change relative to chow as described previously^43^.

For miRNA analysis, TaqMan® microRNA Reverse Transcription Kit (Life Technologies) and a specific miR-21 (002493) TaqMan® probe was used for reverse transcription using 10 ng of total RNA. Real-time PCR was performed in a 7500 Fast Real-Time PCR machine using miRNA TaqMan® probes for miR-21 (002493) and the endogenous control sno-202 (001232). Each sample was run in duplicate and relative miRNA levels were calculated using the comparative Ct (ΔΔCt) method, expressed as fold-change relative to chow.

### Histology and immunohistochemistry

For light microscopic examinations, 3 µm renal paraffin sections were stained with Periodic acid-Schiff (PAS). In short, paraffin sections were deparaffinised and re-hydrated in distilled water. Sections were placed in 0.5% periodic acid solution for 5 minutes. After rinsing in distilled water, sections were incubated in Schiff’s reagent (Sigma-Aldrich, Zwijndrecht, the Netherlands) for 15 minutes, followed by rinsing in lukewarm water for 5 minutes. Sections were counterstained with Mayer’s haematoxylin for 1 minute and washed in tap water. Images were taken with a Leica microscope using QwinV3 software (Qwin V3 software, Leica Microsystems Imaging Solutions Ltd., Cambridge, UK). Immunofluorescence staining of KIM-1 was performed using antibody NBP1-76701 (Novus Biologicals, Abingdon, UK). For direct visualization of collagen fibers in kidney, a trichrome staining was performed using the Masson’s Trichrome Staining kit (Accustain HT15, Sigma‐Aldrich). To quantify renal fibrosis, sections were stained with Picro-Sirius Red for collagen content, and the extent of fibrosis was quantified at 40x magnification using an automated macro in the image processing software ImageJ (version 1.48, NIH, Bethesda, MD, USA). Collagen content was expressed as the percentage of the total tissue area that was positively stained. Glomeruli and vessels larger than the size of adjacent tubules were excluded when assessing the images.

### Kidney function measured by albumin/creatinine ratio

To assess renal function, urinary albumin and creatinine levels were measured using commercially available kits. For this, Mouse Albumin ELISA Quantitation Set (Bethyl laboratories, Montgomery, Tx, USA) and The Creatinine Companion assay (Exocell, Philadelphia, PA, USA) were applied according to the manufacturer’s instructions.

### Statistical analysis

Data were analyzed with Graphpad Prism software (version 5.03, Graphpad Software Inc., La Jolla, California) and SPSS (version 22, IBM, Armonk, New York). Differences between groups at one specific time point were analysed by 1-way ANOVA followed by LSD post-hoc analysis. To determine the correlation between two parameters, Spearman’s correlation coefficients were calculated. A p-value ≤ 0.05 was considered statistically significant. All data are presented as mean ± SD.

## References

[1] Swinburn BA, Caterson I, Seidell JC, James WP (2004). Diet, nutrition and the prevention of excess weight gain and obesity. Public health nutrition.

[2] Hajer GR, van Haeften TW, Visseren FL (2008). Adipose tissue dysfunction in obesity, diabetes, and vascular diseases. European heart journal.

[3] Foster MC (2011). Association of subcutaneous and visceral adiposity with albuminuria: the Framingham Heart Study. Obesity (Silver Spring, Md.).

[4] Ritz E (2003). Albuminuria and vascular damage–the vicious twins. The New England journal of medicine.

[5] Schmieder RE (2014). Mortality and morbidity in relation to changes in albuminuria, glucose status and systolic blood pressure: an analysis of the ONTARGET and TRANSCEND studies. Diabetologia.

[6] Sharma K (2009). The link between obesity and albuminuria: adiponectin and podocyte dysfunction. Kidney international.

[7] Navarro JF, Mora C (2005). Role of inflammation in diabetic complications. Nephrology, dialysis, transplantation: official publication of the European Dialysis and Transplant Association - European Renal Association.

[8] Bluher M (2014). Adipokines - removing road blocks to obesity and diabetes therapy. Molecular metabolism.

[9] Briffa JF, McAinch AJ, Poronnik P, Hryciw DH (2013). Adipokines as a link between obesity and chronic kidney disease. American journal of physiology. Renal physiology.

[10] Sharma K (2008). Adiponectin regulates albuminuria and podocyte function in mice. The Journal of clinical investigation.

[11] Rutkowski JM (2013). Adiponectin promotes functional recovery after podocyte ablation. Journal of the American Society of Nephrology: JASN.

[12] Halberg N (2009). Systemic fate of the adipocyte-derived factor adiponectin. Diabetes.

[13] Ciliberto G, Arcone R, Wagner EF, Ruther U (1987). Inducible and tissue-specific expression of human C-reactive protein in transgenic mice. The EMBO journal.

[14] Kleemann R (2004). Evidence for anti-inflammatory activity of statins and PPARalpha activators in human C-reactive protein transgenic mice *in vivo* and in cultured human hepatocytes *in vitro*. Blood.

[15] Gierman LM (2012). Metabolic stress-induced inflammation plays a major role in the development of osteoarthritis in mice. Arthritis and Rheumatism.

[16] Levine AS, Billington CJ (1998). Do circulating leptin concentrations reflect body adiposity or energy flux?. The American Journal of Clinical Nutrition.

[17] Ukkola O, Santaniemi M (2002). Adiponectin: a link between excess adiposity and associated comorbidities?. Journal of Molecular Medicine (Berlin, Germany).

[18] Chau BN (2012). MicroRNA-21 promotes fibrosis of the kidney by silencing metabolic pathways. Science translational medicine.

[19] Glowacki F (2013). Increased circulating miR-21 levels are associated with kidney fibrosis. PloS one.

[20] Abdin AA, Baalash AA, Hamooda HE (2010). Effects of rosiglitazone and aspirin on experimental model of induced type 2 diabetes in rats: focus on insulin resistance and inflammatory markers. Journal of diabetes and its complications.

[21] Fidan E (2011). The effects of rosiglitazone and metformin on inflammation and endothelial dysfunction in patients with type 2 diabetes mellitus. Acta Diabetologica.

[22] Mattoo V (2005). Metabolic effects of pioglitazone in combination with insulin in patients with type 2 diabetes mellitus whose disease is not adequately controlled with insulin therapy: results of a six-month, randomized, double-blind, prospective, multicenter, parallel-group study. Clinical therapeutics.

[23] Doh FM (2012). The effect of HMG-CoA reductase inhibitor on insulin resistance in patients undergoing peritoneal dialysis. Cardiovascular drugs and therapy/sponsored by the International Society of Cardiovascular Pharmacotherapy.

[24] Ouchi N, Walsh K (2007). Adiponectin as an anti-inflammatory factor. Clinica chimica acta; international journal of clinical chemistry.

[25] Pajvani UB, Scherer PE (2003). Adiponectin: systemic contributor to insulin sensitivity. Current diabetes reports.

[26] Zoccali C (2002). Adiponectin, metabolic risk factors, and cardiovascular events among patients with end-stage renal disease. Journal of the American Society of Nephrology: JASN.

[27] Nawrocki AR (2006). Mice lacking adiponectin show decreased hepatic insulin sensitivity and reduced responsiveness to peroxisome proliferator-activated receptor gamma agonists. The Journal of biological chemistry.

[28] Lee S (2012). Adiponectin abates diabetes-induced endothelial dysfunction by suppressing oxidative stress, adhesion molecules, and inflammation in type 2 diabetic mice. American journal of physiology. Heart and circulatory physiology.

[29] Gaede P, Lund-Andersen H, Parving HH, Pedersen O (2008). Effect of a multifactorial intervention on mortality in type 2 diabetes. The New England journal of medicine.

[30] Lim AI, Tang SC, Lai KN, Leung JC (2013). Kidney injury molecule-1: more than just an injury marker of tubular epithelial cells?. Journal of cellular physiology.

[31] Gansevoort RT, Nauta FL, Bakker SJ (2010). Albuminuria: all you need to predict outcomes in chronic kidney disease?. Current opinion in nephrology and hypertension.

[32] Corna D (2007). Effects of rosuvastatin on glomerular capillary size-selectivity function in rats with renal mass ablation. American Journal of Nephrology.

[33] Verhulst A, D’Haese PC, De Broe ME (2004). Inhibitors of HMG-CoA reductase reduce receptor-mediated endocytosis in human kidney proximal tubular cells. Journal of the American Society of Nephrology: JASN.

[34] van der Tol A (2012). Statin use and the presence of microalbuminuria. Results from the ERICABEL trial: a non-interventional epidemiological cohort study. PloS one.

[35] Wolf G, Schroeder R, Ziyadeh FN, Stahl RA (2004). Albumin up-regulates the type II transforming growth factor-beta receptor in cultured proximal tubular cells. Kidney international.

[36] Rychlik I, Miltenberger-Miltenyi G, Ritz E (1998). The drama of the continuous increase in end-stage renal failure in patients with type II diabetes mellitus. Nephrology, dialysis, transplantation: official publication of the European Dialysis and Transplant Association - European Renal Association.

[37] Liu G (2010). miR-21 mediates fibrogenic activation of pulmonary fibroblasts and lung fibrosis. The Journal of experimental medicine.

[38] Thum T (2008). MicroRNA-21 contributes to myocardial disease by stimulating MAP kinase signalling in fibroblasts. Nature.

[39] Gomez, I. G. *et al*. Anti-microRNA-21 oligonucleotides prevent Alport nephropathy progression by stimulating metabolic pathways. *The Journal of clinical investigation* (2014). 10.1172/JCI75852 PMC4382246 25415439

[40] Zhong X (2013). miR-21 is a key therapeutic target for renal injury in a mouse model of type 2 diabetes. Diabetologia.

[41] Kleemann R (2003). Rosuvastatin Reduces Atherosclerosis Development Beyond and Independent of Its Plasma Cholesterol–Lowering Effect in APOE*3-Leiden Transgenic Mice: Evidence for Antiinflammatory Effects of Rosuvastatin. Circulation.

[42] Tao L (2010). Adiponectin: an indispensable molecule in rosiglitazone cardioprotection following myocardial infarction. Circulation research.

[43] Morrison MC (2015). Mirtoselect, an anthocyanin-rich bilberry extract, attenuates non-alcoholic steatohepatitis and associated fibrosis in ApoE *3Leiden mice. Journal of hepatology.

